# Patterns and dynamics of neutral lipid fatty acids in ants – implications for ecological studies

**DOI:** 10.1186/s12983-017-0221-1

**Published:** 2017-07-13

**Authors:** Félix B. Rosumek, Adrian Brückner, Nico Blüthgen, Florian Menzel, Michael Heethoff

**Affiliations:** 1 Ecological Networks, Technische Universität Darmstadt, Schnittspahnstr. 3, 64287 Darmstadt, Germany; 2 Department of Ecology and Zoology, Federal University of Santa Catarina, Campus Trindade, Florianópolis, 88040-900 Brazil; 3 Institute of Organismic and Molecular Evolution, Johannes Gutenberg-Universität Mainz, Johannes-von-Müller-Weg 6, 55128 Mainz, Germany

**Keywords:** Direct trophic transfer, Lipid metabolism, Dietary routing, Fatty acid biosynthesis, Trophic enrichment, Trophic ecology, Trophic markers, Formicidae, *Formica fusca*, *Myrmica rubra*

## Abstract

**Background:**

Trophic interactions are a fundamental aspect of ecosystem functioning, but often difficult to observe directly. Several indirect techniques, such as fatty acid analysis, were developed to assess these interactions. Fatty acid profiles may indicate dietary differences, while individual fatty acids can be used as biomarkers. Ants are among the most important terrestrial animal groups, but little is known about their lipid metabolism, and no study so far used fatty acids to study their trophic ecology. We set up a feeding experiment with high- and low-fat food to elucidate patterns and dynamics of neutral lipid fatty acids (NLFAs) assimilation in ants. We asked whether dietary fatty acids are assimilated through direct trophic transfer, how diet influences NLFA total amounts and patterns over time, and whether these assimilation processes are similar across species and life stages.

**Results:**

Ants fed with high-fat food quickly accumulated specific dietary fatty acids (C18:2n6, C18:3n3 and C18:3n6), compared to ants fed with low-fat food. Dietary fat content did not affect total body fat of workers or amounts of fatty acids extensively biosynthesized by animals (C16:0, C18:0, C18:1n9). Larval development had a strong effect on the composition and amounts of C16:0, C18:0 and C18:1n9. NLFA compositions reflected dietary differences, which became more pronounced over time. Assimilation of specific dietary NLFAs was similar regardless of species or life stage, but these factors affected dynamics of other NLFAs, composition and total fat.

**Conclusions:**

We showed that ants accumulated certain dietary fatty acids via direct trophic transfer. Fat content of the diet had no effect on lipids stored by ants, which were able to synthesize high amounts of NLFAs from a sugar-based diet. Nevertheless, dietary NLFAs had a strong effect on metabolic dynamics and profiles. Fatty acids are a useful tool to study trophic biology of ants, and could be applied in an ecological context, although factors that affect NLFA patterns should be taken into account. Further studies should address which NLFAs can be used as biomarkers in natural ant communities, and how factors other than diet affect fatty acid dynamics and composition of species with distinct life histories.

**Electronic supplementary material:**

The online version of this article (doi:10.1186/s12983-017-0221-1) contains supplementary material, which is available to authorized users.

## Background

Trophic interactions play a central role in ecosystem processes, shaping complex food webs with multiple paths and levels [1]. The complexity of interactions within communities, however, makes it difficult to assess their nature and long-term outcome solely by field observations. Several complementary approaches were developed to address this issue, such as fatty acid analysis [2]. Fatty acids have been used to study trophic ecology of organisms in aquatic and terrestrial ecosystems [3, 4]. Variation in fatty acid profiles can answer basic questions about spatial and temporal variation in diets, as well as niche partitioning among species [3, 5, 6]. Also, fatty acids could be used as biomarkers, indicating qualitative and quantitative trophic relationships between organisms [7, 8]. Many recent studies using fatty acid analysis in terrestrial organisms focused on detritivores, such as Collembola and Nematoda [7, 9–14], which established the technique as a useful tool to analyze their feeding interactions in soil food webs [5, 15–17]. However, fatty acid patterns and dynamics depend on an organism’s physiology and composition of its natural diet, which are variable among taxonomic groups. Therefore, basic information on lipid metabolism is needed before the application of fatty acid analyses to study trophic relations of a given animal group.

Ants (Hymenoptera: Formicidae) are among the most abundant groups of invertebrates in terrestrial ecosystems, with a wide variety of feeding habits, nesting sites, and interactions with organisms from all trophic levels [18]. Many ant species have a cryptic behavior, which is difficult to study directly (e.g., living underground, inside the leaf-litter or in tree canopies). Moreover, in diverse ecosystems, dozens of species can coexist simultaneously in a given stratum [19]. Thus, complementary techniques are needed to study their trophic ecology. Stable isotopes, for instance, have been extensively used to address many questions in ant ecology [20–22]. The application of DNA barcoding, another modern technique, is still incipient for ants [23–25]. Surprisingly, no study so far tested the applicability of fatty acids to understand trophic ecology of ants.

Ants in general are regarded as omnivorous, feeding on a combination of living prey, dead arthropods, seeds and plant exudates. Less common are specialized feeding habits such as fungus cultivation and predation exclusively upon certain arthropod groups, as well as use of unusual resources such as pollen, animal excrements or mushrooms [18, 26–29]. Fatty acids from the diet could be incorporated without modification (i.e. through direct trophic transfer), or actively modified in response to environmental factors and physiological needs [4, 30, 31]. Many ant species primarily feed on sugars usually obtained from floral and extra-floral nectar or honeydew [32]. Like all higher organisms, they can synthesize a set of fatty acids from carbohydrates via a decarboxylative Claisen condensation [33]. Fatty acids are mainly stored as neutral lipid fatty acids (NLFAs), which mostly consist of triglycerides, the principal component of the insect fat body [30, 34]. The biosynthesis of saturated palmitic (C16:0) and stearic acids (C18:0) and monounsaturated oleic acid (C18:1n9) seems to be widespread among insects, and correspondingly these fatty acids are the most abundant in their bodies [30]. On the other hand, the ability to synthesize polyunsaturated fatty acids, such as linoleic acid (C18:2n6), is highly variable among species [35, 36]. However, the details of these physiological processes in ants are poorly understood, and there are no studies specifically addressing dynamics of dietary fatty acids assimilation in this important insect group. Knowing which fatty acids can be unambiguously related to food sources, and how well the overall fat composition of ants reflects their diet after any metabolic modification, are crucial steps to apply fatty acid analysis in an ecological context.

Considering the potential use of fatty acids to understand trophic relations, and the lack of information about lipid metabolism in ants, we aim to elucidate patterns and dynamics of neutral lipid fatty acids in ants. We provided ants with high- and low-fat food in a no-choice feeding experiment, and compared the fatty acid profiles of ant workers and larvae over a period of 8 weeks. We specifically ask: (1) whether NLFA amounts and compositions are affected by a high- and a low-fat diet; (2) whether dietary fatty acids are accumulated in the ants’ body via direct trophic transfer; (3) how dietary fatty acids shape NLFA patterns over time; (4) whether these patterns and dynamics are the same in different species and life stages.

## Methods

### Studied species

The experiment was performed with colonies reared in the laboratory, during November and December 2016. We chose two species, common and widespread in the Northern hemisphere, which represent the largest Formicidae subfamilies: *Formica fusca* Linnaeus 1758 (Formicinae) and *Myrmica rubra* Linnaeus 1758 (Myrmicinae). Both have in nature a similar and generalized diet of living and dead arthropods, nectar and honeydew [37, 38], and can thus be reared in the laboratory with a single artificial diet. Six colonies of each species were purchased from Antstore (Berlin, Germany) where ants were fed on an unstandardized diet of honey and dead flies. All colonies had one queen and between 9 and 12 (*F. fusca*) and 15–20 (*M. rubra*) workers. Colonies of *M. rubra* were reproductive during the whole experiment, with lower numbers of eggs and larvae towards the end. For *F. fusca*, larvae were only observed in two colonies in the last week of the experiment. Colonies were kept at a constant temperature of 25 °C and provided three times per week with water and food *ad libitum*.

### Low- and high-fat treatments

Three colonies of each species received a low-fat treatment, whereas the remaining three received a high-fat treatment. As low-fat food we used a standardized recipe, suitable for breeding several ant species [39]. It contained 5 g agar, 1 g table salt (NaCl), 1 g vitamin-mineral mix powder (Altapharma, Burgwedel, Germany), 62 ml honey and 1 chicken egg homogenized in 500 ml hot water. The high-fat food followed the same recipe, with addition of 60 ml linseed oil (organic quality, Alnatura, Bickenbach, Germany). The mixture was stirred until it was cool and solid, to avoid separation of the aqueous and fatty phases. Both food mixtures were stored in a freezer at −20 °C until use, and food samples were taken for chemical analysis.

### Experimental design

Before beginning the feeding experiment, we collected one worker per colony for fatty acid analysis (= week 0). Workers were chosen randomly from inside and outside the nest (a glass vial kept inside a plastic box). In addition, one larva of *M. rubra* was collected per colony. After starting to apply the treatments, we sampled one worker and one larva in the same way, every week for 8 weeks. Larva sample sizes were smaller from week 5 onwards, because some colonies were not reproductive anymore. In the last week, we also collected the queens for analysis (6 *F. fusca* and 5 *M. rubra*, since one queen died at the beginning of the experiment). All samples were immediately frozen at −20 °C until extraction.

### Fatty acid analysis

Total lipids were extracted from the ants using 1 ml of a chloroform:methanol mixture, 2:1 (*v*/v) over a period of 24 h [40, 41]. Ants were directly refrozen after extraction and subsequently dried for 48 h at 50 °C and weighed with a microbalance (Mettler Toledo, XS3DU, Columbus, USA). The extracts were purified and separated according to the method described by Frostegård et al. [42]. SiOH-columns (Chromabond®) were washed and conditioned with 6 ml hexane. Subsequently, samples were applied on the column and elution of NLFAs (= mono-, di-, and triglycerides) was accomplished with 4 ml chloroform.

The chloroform fractions were evaporated to dryness under gentle nitrogen gas flow and residuals were redissolved in different concentrations of dichloromethane:methanol 2:1 (*v*/v) to adjust the samples to comparable concentration ranges: 1 ml for *F. fusca* queens and food samples, 350 μl for workers of both species and *M. rubra* queens, and 50 μl for larvae. 50 μl aliquots (10 μl for high-fat food) were transferred to new glass vials with a conical inlet (150 μl) and 20 μl of internal standard (C19:0 in methanol; ρ_i_ = 220 ng/μl) were added. Samples were evaporated to dryness again, and finally derivatized to fatty acid methyl esters (FAMEs) with 20 μl TMSH (trimethylsulfonium hydroxide; 0.25 M in MeOH from Fluka, Sigma-Aldrich, St. Louis, USA).

FAME samples of NLFAs were analyzed with a QP2010 Ultra GC/MS (Shimadzu, Duisburg, Germany). The gas chromatograph (GC) was equipped with a ZB-5MS fused silica capillary column (30 m × 0.25 mm ID, df = 0.25 μm) from Phenomenex (Aschaffenburg, Germany). Sample aliquots of 1 μl were injected by using an AOC-20i autosampler-system from Shimadzu into a PTV-split/splitless-injector (Optic 4, ATAS GL, Eindhoven, Netherlands), which operated in splitless-mode. Injection-temperature was programmed from initial 70 °C up to 300 °C and then an isothermal hold for 59 min, sampling-time was set to 3 min and hydrogen was used as carrier-gas with a constant flow rate of 1.3 ml/min. The temperature of the GC oven was raised from initial 60 °C for 1 min, to 150 °C with a heating-rate of 15 °C/min, to 260 °C with a heating-rate of 3 °C/min, to 320 °C with a heating-rate of 10 °C/min and then an isothermal hold at 320 °C for 10 min. Electron ionization mass spectra were recorded at 70 eV from *m/z* 40 to 650. The transfer line and ion source were kept at 250 °C.

Methyl esters of the NLFAs were identified by comparing gas chromatographic retention times and *m/z* fragmentation patterns with those of the Supelco® 37 Component FAME Mix standard and the Bacterial Acid Methyl Ester (BAME) Mix standards as commercially available fatty acids (all Sigma-Aldrich) and published literature data [31, 43, 44]. The identity of γ-linolenic acid was additionally confirmed by an iodine catalyzed dimethyl disulfide derivatization [45].

A technical problem during analysis resulted in the loss of a batch of samples. Therefore, we have no data of week 3 for *M. rubra* larvae, week 4 for *M. rubra* workers and week 5 for *F. fusca*.

### Data analysis

In general we used two approaches to analyse our data: (1) linear mixed-effect models (LMM) to assess the trophic transfer of certain fatty acids; and (2) multivariate compositional data analysis to describe total NLFA patterns. Only fatty acids with >1% composition were included in our analyses. Queens were not statistically analyzed, since they were sampled just at the end of the experiment.

We used the absolute amount of NLFAs [μg] standardized by dry weight for ants or fresh weight for food [mg], thus reflecting the relative amounts of NLFAs in comparison to non-lipid components [μg/mg]. We additionally ran the analyses with absolute amounts and dry weight as a cofactor, and results were identical for workers, but different for larvae, due to their distinct dynamics (see S1 in Additional file 1, and results for larvae).

At first, we correlated the relative amounts of all NLFAs combined (= total NLFAs) with dry weights of larvae and workers of both species using Spearman’s rank correlation. For adults, body weight reflects size polymorphism among workers. For larvae, body weight is a better indicator of larval development than the week of sampling, because queens lay eggs continuously during the reproductive time. Dry weights for workers did not differ between treatments and over time, while larval dry weight increased over time (see S2 in Additional file 1). Since time and size were correlated for larvae (ρ_S_ = 0.63, *p* < 0.001), we ran separated LMMs for each factor, with dry weight normalized by square-root transformation.

We statistically tested relative amounts of total NLFAs and of the three most abundant fatty acids (C16:0, C18:0, C18:1n9). We also tested a specific dietary NLFA (C18:2n6), which occurred in higher concentration in the high-fat diet, and was not conspicuously synthesized by the ants. We did not test the amounts of the other two specific dietary NLFAs (C18:3n3 and C18:3n6) and show their results only in plots, because both were always zero in the low-fat treatment and non-zero in the high-fat treatment. Remaining NLFAs that occurred only in very small amounts in ants and food and were not tested either.

Effects on relative amounts were tested with linear mixed-effect models (command lme) as implemented in the R package “nlme” [46] with feeding treatment and time as fixed factors and colony ID as random factor for each species separately. We checked for the normal distribution of the residuals and the homogeneity of variance prior to the analyses and transformed the data if necessary (see S3 in Additional file 1 for data transformation). We further investigated the total NLFA amount in *M. rubra* workers and larvae using a LMM with the same structure as before, but including life stage as a further fixed factor. The difference between workers and larvae was analyzed with a simultaneous test for general linear hypothesis using Tukey pairwise contrasts (package “multcomp”; [47]) of the previous LMM.

We furthermore analyzed whether the overall NLFA composition (i.e. percentages of all fatty acids) of *F. fusca*, *M. rubra* workers and *M. rubra* larvae changed in the different treatments over time. We tested Bray-Curtis similarities (BCS) based on compositional data using permutational multivariate analysis of variance (PERMANOVA; [48]) for each species separately. Overall 10,000 permutations were performed with feeding treatment and time as fixed factors and colony ID as random factor. We checked the multivariate homogeneity of group dispersions before with a multivariate Levene’s test (PERMDISP; all *p* values >0.1; [49]). These analyses were performed with PRIMER 7.0.12 [50].

Finally, NLFA compositional data were ordinated using principal component analyses (PCA) and according PCA biplots. We compared the differences of the overall NLFA composition in *F. fusca* and *M. rubra* who received the high-fat diet during the experimental time. We used the centered log-ratio transformation after replacing zero values to deal with the constant sum constraint of compositional data and make it suitable for PCA (R packages “zCompositions” and “compositions” [51, 52]). PCA biplots were constructed by plotting factor loadings of compounds that significantly contributed (*p* < 0.01) to the group separation onto the PCA scatter plots using the R package “vegan” [53]. For a detailed R script of this analysis, see [54]. LMMs and PCAs were performed with R version 3.3.1 [55].

## Results

### Fatty acid profiles of food and ants

The neutral lipid fatty acid (NLFA) profiles of ants and their food are summarized in Table 1 (for full dataset and value ranges, see Additional file 2). The high-fat food had about 40 times more total concentration of NLFAs than the low-fat food. The main component of the high-fat food was C18:3n3, but it also had notably higher amounts of C16:0, 18:0 and C18:2n6. Besides, it contained C:18:3n6, which was entirely absent from the low-fat food.

**Table 1:** – Fatty acid profiles of food and ants at the beginning and end of the experiment

	*Formica fusca*						*Myrmica rubra*						*Myrmica rubra* larvae				Food	
NLFA	Week 0		Week 8		Queens		Week 0	Week 8			Queens		Week 0	Week 8				
	+	-	+	-	+	-	+	-	+	-	+	-	+	-	+	-	+	-
C12:0 lauric	0.1 (t)	0.1 (t)	0.1 (t)	0.1 (t)	0.1 (t)	0.1 (t)	0.6 (t)	0.4 (t)	0.2 (t)	0.3 (t)	0.1 (1)	0.2 (t)	3.9 (t)	2.4 (t)	0.4 (t)	1.0 (t)	t (t)	t (t)
C14:0 mystric	0.5 (t)	0.3 (t)	0.3 (t)	0.6 (t)	0.1 (t)	0.3 (t)	1.9 (1)	1.6 (t)	0.6 (1)	0.5 (1)	0.1 (1)	0.4 (1)	8.8 (1)	5.0 (1)	1.2 (1)	2.7 (1)	t (t)	t (t)
C16:0 palmitic	59.0 (25)	40.6 (25)	95.2 (12)	129.8 (18)	25.5 (11)	30.5 (18)	55.7 (18)	54.0 (18)	23.1 (32)	31.3 (30)	3.0 (26)	10.3 (17)	532.7 (54)	397.1 (59)	48.9 (33)	50.1 (25)	9.2 (8)	0.8 (32)
C16:1n9 palmitotelic	4.1 (1)	0.9 (t)	2.6 (t)	4.6 (1)	0.9 (t)	2.4 (1)	6.7 (2)	2.6 (1)	1.1 (1)	1.8 (1)	0.1 (1)	1.6 (3)	11.3 (1)	2.7 (t)	1.2 (1)	8.1 (4)	0.1 (t)	t (1)
C18:0 stearic	11.6 (10)	11.4 (12)	26.5 (3)	22.3 (3)	7.2 (3)	5.3 (3)	13.1 (5)	17.4 (6)	7.6 (15)	7.7 (13)	1.7 (15)	1.3 (3)	242.2 (25)	209.2 (31)	29.8 (20)	23.4 (12)	6.6 (6)	0.1 (4)
C18:1n9 oleic	284.7 (64)	224.6 (62)	523.7 (67)	570.3 (78)	151.4 (66)	135.4 (77)	273.5 (74)	251.7 (74)	80.0 (48)	129.2 (55)	4.4 (26)	46.1 (76)	202.6 (19)	51.5 (8)	39.4 (27)	112.2 (56)	1.8 (2)	1.5 (59)
C18:2n6 linoleic	0.4 (t)	0.4 (t)	4.2 (1)	0.2 (t)	3.0 (1)	0.5 (t)	1.3 (1)	1.6 (1)	2.4 (2)	0.3 (t)	1.1 (7)	0.4 (1)	3.2 (t)	2.1 (t)	3.4 (2)	1.3 (1)	5.3 (4)	0.1 (2)
C18:3n3 α-linolenic	0 (0)	0 (0)	138.8 (17)	0 (0)	30.8 (14)	0 (0)	0 (0)	0 (0)	3.9 (2)	0 (0)	3.1 (18)	0 (0)	0 (0)	0 (0)	20 (14)	0 (0)	80.9 (72)	t (1)
C18:3n6 γ-linolenic	0 (0)	0 (0)	22.5 (3)	0 (0)	9.1 (4)	0 (0)	0 (0)	0 (0)	1.3 (1)	0 (0)	0.9 (5)	0 (0)	0 (0)	0 (0)	1.7 (1)	0 (0)	8.9 (8)	0 (0)
C20:0 arachidic	0.1 (t)	0.1 (t)	0.1 (t)	0.1 (t)	0.1 (t)	0 (t)	0.2 (t)	0.2 (t)	0.1 (t)	0.1 (t)	0.1 (t)	0.1 (t)	0.7 (t)	0.6 (t)	0.2 (t)	0.1 (t)	t (t)	t (t)
Total	360.3	278.4	813.9	728.0	228.0	174.5	353.1	329.7	120.3	171.2	14.5	60.3	1005.5	670.6	146.3	198.9	112.8	2.6
Sample size	3	3	3	3	3	3	3	3	3	3	2	3	3	3	1	1	3	2

Average amounts are given in μg of NLFA/mg of dry weight (fresh weight for food). Values in brackets are average percentages of the total composition of NLFAs per sample. +: high-fat treatment; −: low-fat treatment; t: detected in trace amount (less than 0.1 μg/mg or 1% of composition)

C16:0, C18:0 and C18:1n9 were the main fatty acids in ants (Table 1). C18:1n9 was the main component in all experimental workers and queens. On the other hand, larvae had comparatively high levels of C16:0 and C18:0. Ants from the high-fat treatment exhibited higher amounts of C18:2n6, and were the only ones with detectable levels of C18:3n3 and C18:3n6. Queens had less total NLFAs than workers. Samples were variable, thus the profiles in Table 1 do not exactly reflect temporal and treatment differences (particularly for the highly variable larvae); these effects are analyzed below.

### Dynamics of total and individual NLFA amounts

**Fig. 1:**
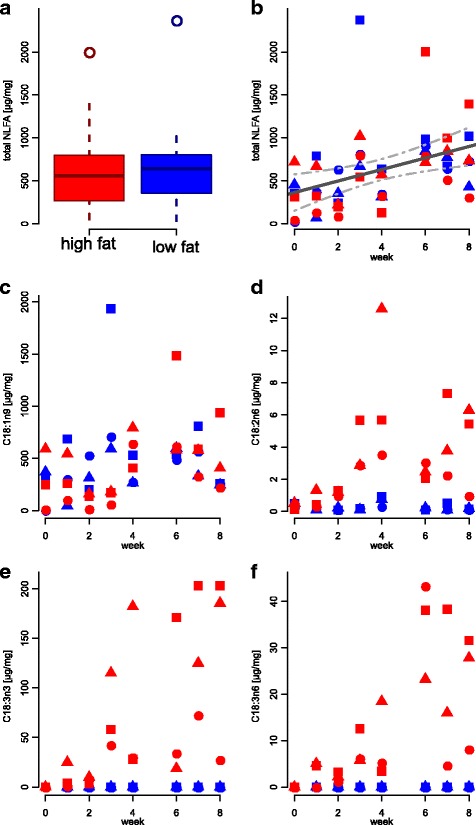
Dynamics of NLFA total amount and individual fatty acids in *Formica fusca* workers. Symbols indicate distinct colonies. In (**a**) samples from all weeks and colonies are pooled, (**b**) total NLFA, (**c**) C18:1n9, (**d**) C18:2n6, (**e**) C18:3n3, (**f**) C18:3n6

For *F. fusca*, there was no difference between treatments in the total amount of NLFAs (Fig. 1a, Table 2). C16:0, C18:0, C18:1n9 and total NLFAs increased over time, but with no treatment effect (Fig. 1b-c, Table 2). On the other hand, we observed an increasingly higher amount of C18:2n6 in the high-fat treatment, while it remained small in the low-fat treatment (Fig. 1d, Table 2). Similarly, C18:3n3 and C18:3n6 increased remarkably in the high-fat treatment, but were never recorded in the low-fat treatment (Figs. 1e-f). *Formica fusca* presented considerable polymorphism (coefficient of variation [= CV] of dry weights = 41%), but there was no correlation between body size and total NLFA amount (ρ_S_ = −0.06, *p* = 0.66).

**Table 2:** Effects of time, treatment and larval dry weight on relative total amount [μg/mg] and individual amounts of fatty acids

	Total NLFAs				C16:0				C18:0				C18:1n9				C18:2n6			
	df	F	trend	p	df	F	trend	p	df	F	trend	p	df	F	trend	p	df	F	trend	p
*F. fusca* (*n* = 48)																				
Treatment	1	0.12		0.74	1	1.39		0.303	1	0.80		0.422	1	0.18		0.69	1	58.44		**0.002**
Time	1	15.42	**↑**	**< 0.001**	1	22.30	**↑**	**< 0.001**	1	25.51	**↑**	**< 0.001**	1	10.52	**↑**	**0.002**	1	11.66		**0.002**
Treatment x Time	1	0.29		0.60	1	0.16		0.687	1	1.94		0.171	1	0.84		0.37	1	14.97	**↑**_**high**_	**< 0.001**
Residuals	44				44				44				44				44		**↓**_**low**_	
*M. rubra* (*n* = 48)																				
Treatment	1	0.02		0.90	1	0.59		0.484	1	0.02		0.893	1	0.26		0.64	1	29.06	**↑**	**0.006**
Time	1	6.34	**↓**	**0.016**	1	4.60	**↓**	**0.038**	1	9.85	**↓**	**0.003**	1	4.36	**↓**	**0.043**	1	2.98		0.09
Treatment x Time	1	0.07		0.79	1	0.23		0.634	1	0.01		0.956	1	0.02		0.89	1	2.12		0.15
Residuals	44				44				44				44				44			
*M. rubra* larvae [A] (*n* = 38)																				
Treatment	1	11.08	**↑**	**0.029**	1	1.36		0.308	1	3.20		0.148	1	9.93	**↑**	**0.034**	1	22.55	**↑**	**0.009**
Time	1	12.27	**↓**	**0.015**	1	9.62	**↓**	**< 0.001**	1	37.08	**↓**	**< 0.001**	1	0.46		0.502	1	0.09		0.765
Treatment x Time	1	0.34		0.56	1	0.20		0.656	1	2.64		0.115	1	1.23		0.276	1	2.17		0.151
Residuals	33				33				33				33				33			
*M. rubra* larvae [B] (*n* = 38)																				
Treatment	1	25.78	**↑**	**< 0.001**	1	2.05		0.161	1	2.75		0.107	1	10.80		**0.003**	1	21.57	**↑**	**0.001**
Dry weight	1	46.35	**↓**	**< 0.001**	1	53.59	**↓**	**< 0.001**	1	69.50	**↓**	**< 0.001**	1	0.15		0.700	1	0.62		0.434
Treatment x Dry weight	1	3.36		0.076	1	1.42		0.241	1	0.05		0.809	1	4.68	**↑**_**low**_	**0.038**	1	0.06		0.802
Residuals	33				33				33				33		**↓**_**high**_		33			

Results of linear mixed-effect models. Trends indicate the direction of significant effects (α < 0.05, in bold). For larvae, [A] = time as a factor, [B] = dry weight as a factor

**Fig. 2:**
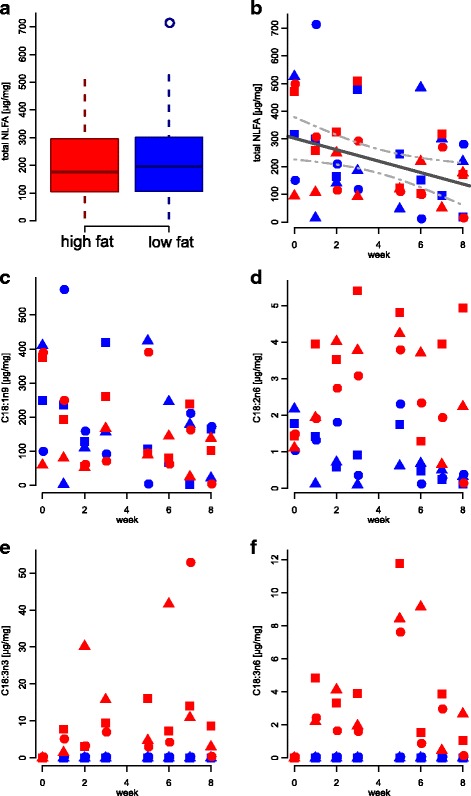
Dynamics of NLFA total amount and individual fatty acids in *Myrmica rubra* workers. Symbols indicate distinct colonies. In (**a**) samples from all weeks and colonies are pooled, (**b**) total NLFA, (**c**) C18:1n9, (**d**) C18:2n6, (**e**) C18:3n3, (**f**) C18:3n6

For *M. rubra*, the amounts of C18:2n6, C18:3n3 and C18:3n6 also increased in the high-fat treatment, and the last two NLFAs were completely absent in the low-fat treatment (Fig. 2d-f, Table 2). No time effect was observed for C18:2n6 in this species. There was no treatment effect in C16:0, C18:0, C18:1n9 and total NLFAs, but, opposite to *F. fusca*, we observed an overall decrease over time (Figs. 2a-b, Table 2). *Myrmica rubra* workers varied less in size (CV of dry weights = 17%) and, again, no correlation was found between body size and total NLFA amount (ρ_S_ = 0.19, *p* = 0.19).

**Fig. 3:**
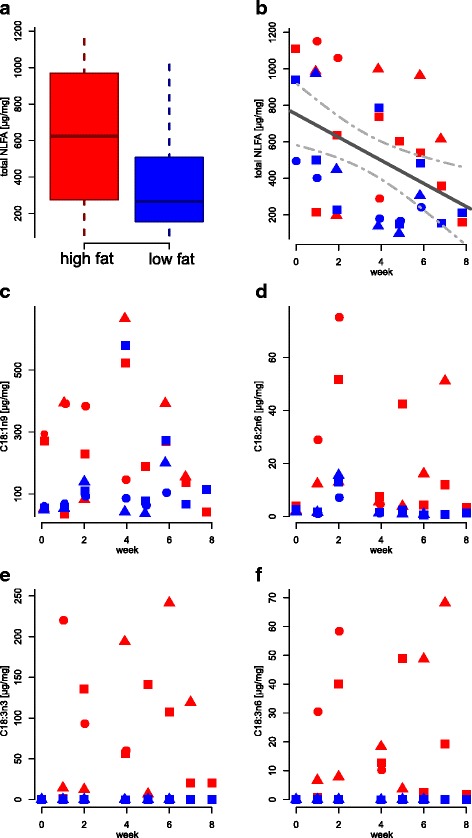
Dynamics of NLFA total amount and individual fatty acids in *Myrmica rubra* larvae. Symbols indicate distinct colonies. In (**a**) samples from all weeks and colonies are pooled

**Fig. 4:**
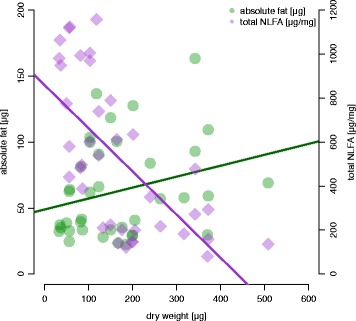
Effects of increasing dry weight on absolute and relative NLFA amounts of *Myrmica rubra* larvae

*Myrmica rubra* larvae presented more complex dynamics, because they were influenced both by experimental time effect and their developmental stage. Nevertheless, since these variables were correlated, LMM results were similar, except for C18:1n9 (Table 2). The increasing trends for C18:2n6, C18:3n3 and C18:3n6 were the same as in workers (Fig. 3d-f, Table 2). Total NLFAs also decreased with time (Fig. 3b, Table 2), but in a higher rate than in workers (Tukey pairwise contrasts, z = 4.70, *p* < 0.001, for full model see S4 in Additional file 1). Larvae from the high-fat treatment had more total NLFAs and C18:1n9 overall during the experiment (Figs. 3a-c, Table 2 [A]). However, as larvae increased in dry weight, C18:1n9 actually was higher in the low-fat treatment compared to the high-fat treatment (Table 2 [B]). There was a strong negative correlation between larval dry weight and relative NLFA amount (Fig. 4; Table 2 [B], ρ_S_ = −0.72, *p* < 0.001). The absolute amount of fat slightly increased with body size, but did not follow the growth in other body components, which resulted in lower concentration of NLFAs in larger and older larvae (Fig. 4). This decrease was mostly due to a decline on saturated fatty acids (C16:0 and C18:0, Table 2, see S5 in Additional file 1). Therefore, young larvae had relatively large fat storages and high ratios of saturated:unsaturated fatty acids, which both decreased during development.

### Dynamics of overall fatty acid composition

**Fig. 5:**
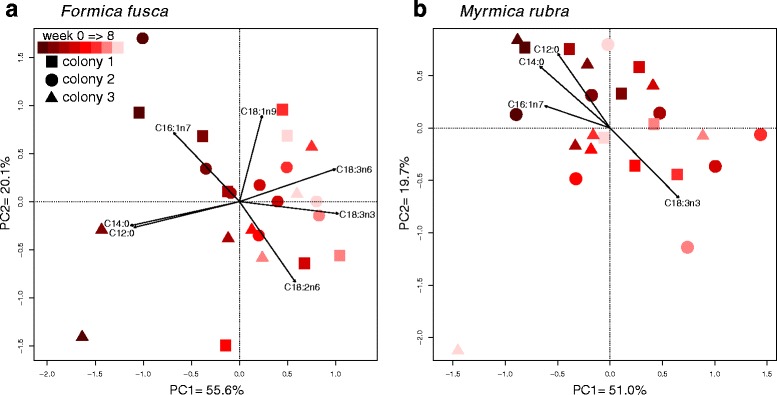
Principal component analysis for changes in composition over time of colonies under the high-fat treatment. Lighter colors mean later weeks. Arrows show fatty acids with significant effects over compositional changes (for factor loadings, see S6 in Additional file 1). (**a**) *Formica fusca*, (**b**) *Myrmica rubra*

The overall NLFA composition of the ants changed over time (Table 3). Treatment and time affected the composition of *F. fusca* and *M. rubra* larvae. For *M. rubra* workers, no effect was found. However, this could be understood when the profile change of the high-fat colonies was analyzed with PCA (Fig. 5). For both species, we noticed a shift in composition over time, mainly driven by the dietary fatty acids. For *F. fusca*, C18:2n6, C18:3n3 and C18:3n6 altogether had a statistically significant effect on this shift. For *M. rubra*, only C18:3n3 (the main dietary fatty acid) had a significant effect. The samples from week 8 were particularly odd, showing small proportions of C18:3n3 and C18:1n9 and relatively high proportions of C16:0 and C18:0. One individual from each treatment had unusually low amounts of total fat and oleic acid (below 20 μg/mg and 10% of composition, respectively; see Additional file 2), which added significant variation to the results. When week 8 was removed from the PERMANOVA, the treatment effect was noticeable (Table 3).

**Table 3:** Effects of time and treatment on overall NLFA composition

	df	pseudoF	p
*F. fusca*			
Treatment	1	8.87	**< 0.001**
Time	7	2.60	**0.015**
Treatment x time	7	0.91	0.543
Residuals	32		
*M. rubra* (week 8)			
Treatment	1	2.98	0.089
Time	7	1.44	0.197
Treatment x time	7	0.53	0.832
Residuals	32		
*M. rubra* (week 7)			
Treatment	1	4.70	**0.025**
Time	6	1.46	0.193
Treatment x time	6	0.87	0.871
Residuals	28		
*M. rubra* larvae			
Treatment	1	22.46	**< 0.001**
Time	7	12.45	**< 0.001**
Treatment x time	7	1.76	0.090
Residuals	23		

PERMANOVA results for overall composition (%) based on Bray-Curtis Similarities. Significant results (*p* < 0.05) are in bold

## Discussion

### Fatty acid profiles of ants

Several factors influence the fatty acid composition of insects, such as flying activity, life stage, growth, reproductive status, environmental temperature, and diet [4, 30, 56]. Due to this complexity, Stanley-Samuelson et al. [30] argued against a “typical” insect profile, and indeed a high variation is found among orders, families, and species [56, 57]. Just a few ant profiles are available in literature: *Myrmica incompleta* Provancher, 1881 (worker and pupae; [58]), *Lasius claviger* (Roger, 1862) (only pupae; [59]), *Myrmica rubra* (only the free fatty acid fraction from head extracts; [60, 61]) and *Polyrhachis dives* Smith, 1857 (sun-dried workers cultivated as food; [62]). These fatty acid profiles are not entirely comparable due to the multitude of goals and methods, but, together with our results, they indicate C18:1n9 as the predominant NLFA in ant bodies, followed by C16:0 and C18:0. High levels of C18:1n9 are standard for Hymenoptera, but the abundance of other fatty acids varies within the order [56].

### Dynamics of individual NLFAs and overall composition

Some fatty acids are extensively synthesized de novo by animals, while others are produced in small amounts, or only by certain taxa [30, 36]. In our experiment, the food enrichment with linseed oil allowed us to observe the influence of diet on NLFAs found a priori in high, low and null amounts in ants’ bodies. C18:3n3 and C18:3n6 were absent in week 0, and solely recorded in the high-fat treatment during the experiment. This suggests that ants are not able to synthesize them, or only in small doses which are directly incorporated in the polar lipid fractions (i.e. phospholipids, glycolipids, free fatty acids). The amounts of C18:3n3 and C18:3n6 increased with the time ants fed on the diet, thus their concentration reflects how much/how often the ants consumed a resource. If these NLFAs are neither highly mobilized nor modified, they should mainly be stored in the fat body when acquired in considerable amounts from the diet, and thus detectable with neutral lipid fatty acid analysis.

C18:2n6 was found in smaller amounts in all samples of the low-fat treatment, but it is not clear whether this fatty acid was synthesized by ants de novo, was obtained from the small amounts in the food, or from the pre-experimental diet. About one third of reported insect species, from five different orders, are able to synthesis C18:2n6, but high interspecific variation was observed within orders [35, 36]. Regarding the Hymenoptera, C18:2n6 biosynthesis was not observed in the mason bee *Osmia lignaria* Say, 1837 (Megachilidae) [35], but it is known from the parasitoid *Nasonia vitripennis* (Walker, 1836) (Pteromalidae) [63]. Regardless of the actual ability of ants to synthesize C18:2n6, its amounts also increased with the diet and, in *F. fusca*, over time as well. In *M. rubra* and its larvae the time effect was not clear.

On the other hand, C16:0, C18:0 and C18:1n9 behaved similarly in both treatments. No treatment effect in C16:0 and C18:0 was noticed, even if they occurred in the high-fat food in levels higher than C18:3n6 and C18:2n6, respectively. Hence, it seems most likely that C16:0, C18:0 and C18:1n9 are synthesized de novo in large amounts from carbohydrates and constantly modified depending on physiological requirements. For example, the physiologically ideal fluidity of the fat body, which changes accordingly with environmental temperature, is achieved through a balanced ratio between saturated and unsaturated fatty acids [4]. Hence, the interplay between β-oxidation and Claisen condensation of these abundant NLFAs should be essential for this mechanism. The lack of a treatment effect on total NLFAs also suggests that, at least under *ad libitum* feeding conditions, ants have no significant energetic loss due to de novo fatty acid biosynthesis. Thus, ants with a sugar-based diet should not have a disadvantage compared to species that acquire most lipids from the diet. However, this may not be true under conditions with limited resources, and detectable differences in ratios could occur between ants that feed directly on lipids and ants that only synthesize them.

Our multivariate analyses showed that a shift in diet results in an equivalent shift in profile, and this difference was more pronounced when the ants fed longer on that resource (Table 3, Fig. 5). The main drivers of this compositional change were specific dietary NLFAs. Therefore, these profiles represent another way to assess dynamics of resource use or detect differences among species [3, 5, 6]. They could be particularly useful when the exact lipid composition of the food is not known, such as in samples collected from the field.

### Factors affecting NLFA dynamics

Our results point out to several factors that affect lipid metabolism in ants, and could be important from biological and methodological points of view. First of all, one possible caveat of analytical methods that use ants’ full body is that the undigested food stored in their crops could bias the results [20]. If this were the case in our experiment, we would expect higher total amount of lipids in ants of the high-fat treatment, and a conspicuous increase during the first week. Also, higher variance should occur in the high-fat treatment, due to the collection of workers with variable crop filling. However, (1) the amount of NLFAs did not differ between treatments, with the exception of larvae (which do not possess a crop; [64]); (2) we observed linear patterns for total fat and several NLFAs, consistent with lipid storage in the fat body; and (3) variances did not differ between treatments, in all cases (F test; *F. fusca* – F = 1.01, *p* = 0.97, *M. rubra* – F = 1.58, *p* = 0.28, larvae – F = 1.67, *p* = 0.28). Even if ants had undigested food in their crops, its contribution would have been relatively small. Thus, as far as the dietary component of interest does not occur in very high amounts in the food (e.g. ca. 10% NLFAs in our high-fat diet), full body extraction can be used to investigate the effect of diet in ants. In certain research contexts, however, it might be important to fully eliminate this factor, using a methodological alternative such as dissection of the fat body.

The reproductive status of the colonies influenced fatty acid dynamics. Feeding the brood can negatively affect the amount of fat stored by the workers, as observed in *Camponotus festinatus* (Buckley, 1866) [65], potentially explaining the decrease of NLFAs in *M. rubra*. On the contrary, *F. fusca* colonies were getting closer to reproduction mode during the experiment, and effectively we observed larvae in two colonies at the last week (this reproductive timing was also observed in non-experimental colonies kept in the same conditions). These colonies needed to accumulate reserves to fuel upcoming larval feeding and egg laying. Considering this, it is intriguing that queens of both species displayed a very low amount of fat at the end of the experiment.

We also observed an effect of development in compositions and dynamics of *M. rubra* larvae. The young larvae had large fat storages and amounts of saturated fatty acids. Earlier in their growth process, they quickly develop other tissues to build more complex organs [66], resulting in a proportionally smaller amount of NLFAs. The increase in C18:1n9 in the low-fat treatment with development may appear counterintuitive, but this was the only unsaturated NLFA ants were able to synthesize in large amounts. In turn, larvae from the high-fat treatment already received several polyunsaturated NLFAs from the diet. The shift to a more balanced composition between saturated and unsaturated NLFAs might enhance metabolic processes in a more complex body. In contrat to workers, larvae seem to benefit from a high-fat diet from which they accumulate slightly more NLFAs. For *Solenopsis invicta* Buren, 1972 it has been demonstrated that sugars, lipids and proteins are differently allocated among worker subcastes, larvae and queens [67].

The distinct distribution of nutrients among individuals of a colony is not restricted to different life stages, but also among worker subcastes. Several studies observed higher fat storage in workers that stay inside the nest and take care of the brood (= nurses), and less in workers that spend more time in activities outside the nest (= foragers) [67, 68]. However, this pattern may not occur in a few species, and no difference was previously found in field samples of *F. fusca* [69]. In *M. rubra*, nurse and forager subcastes were identified in laboratory colonies smaller than ours, and their role was related to individual age and size [70]. Differences in worker size were unrelated to total amount of fat for both species in our data. Individual variation in fat storages could indicate behavioral subcastes, but it was the same in the reproductive *M. rubra* and the non-reproductive *F. fusca* (CV of NLFA total amounts for all samples = 72% in both species). Thus, we found no evidence for considerable differences in lipid storage across behavioral or morphological subcastes within these species, under our experimental conditions, although these effects may be minute in small colonies and need a specific setup to be detected.

Regardless of the variation across species and life stages in profiles and dynamics, the assimilation of specific dietary NLFAs (C18:2n6, C18:3n3 and C18:3n6) followed the same pattern. Thus, the physiological processes involved in NLFA metabolism should be conserved at least between the subfamilies Formicinae and Myrmicinae, which comprise about three quarters of all valid ant species [71]. It is likely that all ants behave similarly, but this needs to be tested with experiments using species with more diversified feeding behaviors and from more distant branches of the ant tree of life, such as the Ponerinae or Dorylinae [72].

### Implications to the study of ant trophic ecology

In trophic ecology, fatty acids can basically be used in two ways: as overall profiles, whose variation indicates differences in diet; and as biomarkers, which indicate specific interactions between organisms [3, 4]. Our results suggest that both applications are suitable for ants. Profiles and individual NLFAs observed in ants changed in response to diet, and these shifts became more pronounced over time. Fatty acid analysis can provide a better resource resolution than stable isotopes, in a more quantitative way and representative timeframe than barcoding of gut DNA [2]. However, these methods are complementary, rather than opposing, and could be coupled with field observations to provide a comprehensive perspective on ant trophic ecology.

The factors affecting NLFA amounts and composition that we observed should also be considered in an ecological context. A representative sample of castes and life stages is recommended if one is interested in detailed trophic ecology of a particular species. For a study at community level, profiles of forager workers sampled at a similar time may be enough to provide comparative information on resource partitioning, although distinct reproduction times could influence amounts and compositions.

In this study, we did not aim to survey prospective biomarkers for natural resources used by ants. However, the three specific dietary NLFAs (C18:2n6, C18:3n3 and C18:3n6) presented chemical properties of suitable biomarkers, as they were not produced by ants (or only in small amounts) and assimilated through direct trophic transfer, with little or no metabolic modification [4]. They can be found in natural diets of ants, such as in elaiosomes, seeds and other insects, in variable patterns that may allow detection of specific interactions [56, 73, 74]. Thus, they are good candidates for trophic markers. Since their assimilation was not affected by species identity, reproductive status or life stage, the biomarker approach seems to be quite promising for ants. Naturally, the actual relevance of these NLFAs would depend on context and occurrence within a community. On the other hand, since C16:0, C18:0 and C18:1n9 are synthesized from carbohydrates in large amounts, and highly modified to attend physiological needs, it would be difficult to relate their amounts to a particular resource or feeding behavior. Further research can provide more fatty acids useful as biomarkers, related to other resources used by ants, which would likely be distinct from the ones suggested for other groups (e.g. C18:1n9 as an indicator of herbivory in Collembola [5]).

## Conclusions

We showed that ants accumulated fatty acids from their diet via direct trophic transfer, and that both, individual NLFAs and overall profiles reflect their diets. The fat content of the diet had no effect in lipids stored by ants, which shows that they are able to synthesize large amounts of NLFAs from sugars. Other factors such as reproductive status and life stage also affected total amounts and profiles of NLFAs. Specific dietary fatty acids were assimilated independent of species or life stage. Fatty acid analysis is a suitable technique to study feeding behavior of ants, and can become a valuable tool to study ant trophic ecology in the field. To this end, central points to be addressed by future research are which biomarkers are most informative of ant diets in natural communities, and how factors other than diet affect fatty acid dynamics and composition of ant species with distinct life histories.

## Additional files

Additional file 1: Supplementary information. (PDF 511 kb)

Additional file 2: Full dataset. (XLSX 42 kb)

## References

[1] Polis GA, Strong DR (1996). Food web complexity and community dynamics. Am Nat.

[2] Birkhofer K, Bylund H, Dalin P, Ferlian O, Gagic V, Hambäck PA (2017). Methods to identify the prey of invertebrate predators in terrestrial field studies. Ecol Evol.

[3] Budge SM, Iverson SJ, Koopman HN (2006). Studying trophic ecology in marine ecosystems using fatty acids: a primer on analysis and interpretation. Mar Mammal Sci.

[4] Ruess L, Chamberlain PM (2010). The fat that matters: soil food web analysis using fatty acids and their carbon stable isotope signature. Soil Biol Biochem.

[5] Ruess L, Schütz K, Haubert D, Häggblom MM, Kandeler E, Scheu S (2005). Application of lipid analysis to understand trophic interactions in soil. Ecology.

[6] Ferlian O, Scheu S, Pollierer MM (2012). Trophic interactions in centipedes (Chilopoda, Myriapoda) as indicated by fatty acid patterns: variations with life stage, forest age and season. Soil Biol Biochem.

[7] Chamberlain PM, Bull ID, Black HIJ, Ineson P, Evershed RP (2006). Collembolan trophic preferences determined using fatty acid distributions and compound-specific stable carbon isotope values. Soil Biol Biochem.

[8] Pollierer MM, Scheu S, Haubert D (2010). Taking it to the next level: Trophic transfer of marker fatty acids from basal resource to predators. Soil Biol Biochem.

[9] Ruess L, Häggblom MM, Garcia Zapata EJ, Dighton J (2002). Fatty acids of fungi and nematodes – possible biomarkers in the soil food chain?. Soil Biol Biochem.

[10] Chamberlain PM, Bull ID, Black HIJ, Ineson P, Evershed RP (2005). Fatty acid composition and change in Collembola fed differing diets: identification of trophic biomarkers. Soil Biol Biochem.

[11] Haubert D, Häggblom MM, Scheu S, Ruess L (2004). Effects of fungal food quality and starvation on the fatty acid composition of *Protaphorura fimata* (Collembola). Comp Biochem Physiol B Biochem Mol Biol.

[12] Haubert D, Langel R, Scheu S, Ruess L (2005). Effects of food quality, starvation and life stage on stable isotope fractionation in Collembola. Pedobiologia.

[13] Haubert D, Häggblom MM, Scheu S, Ruess L (2008). Effects of temperature and life stage on the fatty acid composition of Collembola. Eur J Soil Biol.

[14] Haubert D, Pollierer MM, Scheu S (2011). Fatty acid patterns as biomarker for trophic interactions: changes after dietary switch and starvation. Soil Biol Biochem.

[15] Ruess L, Schütz K, Migge-Kleian S, Häggblom MM, Kandeler E, Scheu S (2007). Lipid composition of Collembola and their food resources in deciduous forest stands – implications for feeding strategies. Soil Biol Biochem.

[16] Haubert D, Birkhofer K, Fließbach A, Gehre M, Scheu S, Ruess L (2009). Trophic structure and major trophic links in conventional versus organic farming systems as indicated by carbon stable isotope ratios of fatty acids. Oikos.

[17] Ngosong C, Raupp J, Scheu S, Ruess L (2009). Low importance for a fungal based food web in arable soils under mineral and organic fertilization indicated by Collembola grazers. Soil Biol Biochem.

[18] Kaspari M, Agosti D, Majer JD, Alonso LE, Schultz TR (2000). A primer on ant ecology. Ants: standard methods for measuring and monitoring biodiversity.

[19] Agosti D, Majer J, Alonso L, Schultz T (2000). Sampling ground-dwelling ants: case studies from the worlds’ rain forests.

[20] Blüthgen N, Gebauer G, Fiedler K (2003). Disentangling a rainforest food web using stable isotopes: dietary diversity in a species-rich ant community. Oecologia.

[21] Davidson DW, Cook SC, Snelling RR, Chua TH (2003). Explaining the abundance of ants in lowland tropical rainforest canopies. Science.

[22] Feldhaar H, Gebauer G, Blüthgen N. Stable isotopes: past and future in exposing secrets of ant nutrition (Hymenoptera: Formicidae). Myrmecol. News. 2010;13:3–13.

[23] Fournier V, Hagler J, Daane K, de León J, Groves R (2008). Identifying the predator complex of *Homalodisca vitripennis* (Hemiptera: Cicadellidae): a comparative study of the efficacy of an ELISA and PCR gut content assay. Oecologia.

[24] Muilenburg VL, Goggin FL, Hebert SL, Jia L, Stephen FM (2008). Ant predation on red oak borer confirmed by field observation and molecular gut-content analysis. Agric For Entomol.

[25] Penn HJ, Chapman EG, Harwood JD (2016). Overcoming PCR inhibition during DNA-based gut content analysis of ants. Environ Entomol.

[26] Blüthgen N, Feldhaar H, Lach L, Parr CL, Abbott KL (2010). Food and shelter: how resources influence ant ecology. Ant Ecolgy.

[27] Brandão CRF, Silva RR, Delabie JHC. Neotropical ants (Hymenoptera) functional groups: nutritional and applied implications. In: Panizzi AR, Parra JRP, editors. Insect bioecology and nutrition for integrated Pest management. Boca Raton: CRC Press; 2012. p. 213–36.

[28] von Beeren C, Mair MM, Witte V. Discovery of a second mushroom harvesting ant (hymenoptera: Formicidae) in Malayan tropical rainforests. Myrmecol News. 2014;20:37–42.

[29] Sainz-Borgo C. Bird feces consumption by fire ant *Solenopsis geminata* (Hymenoptera: Formicidae). Entomol News. 2015;124:295–9.

[30] Stanley-Samuelson DW, Jurenka RA, Cripps C, Blomquist GJ, de Renobales M (1988). Fatty acids in insects: composition, metabolism, and biological significance. Arch Insect Biochem Physiol.

[31] Brandstetter B, Ruther J. An insect with a delta-12 desaturase, the jewel wasp *Nasonia vitripennis*, benefits from nutritional supply with linoleic acid. Sci Nat. 2016;103 10.1007/s00114-016-1365-0 27116611

[32] Davidson DW, Cook SC, Snelling RR (2004). Liquid-feeding performances of ants (Formicidae): ecological and evolutionary implications. Oecologia.

[33] Heath RJ, Rock CO (2002). The Claisen condensation in biology. Nat Prod Rep.

[34] Fast PG (1964). Insect lipids: a review. Mem Entomol Soc Can.

[35] Cripps C, Blomquist GJ, de Renobales M (1986). De novo biosynthesis of linoleic acid in insects. Biochim Biophys Acta BBA-Lipids Lipid Metab.

[36] Renobales M, Cripps C, Stanley-Samuelson DW, Jurenka RA, Blomquist GJ (1987). Biosynthesis of linoleic acid in insects. Trends Biochem Sci.

[37] Collingwood CA. The Formicidae (Hymenoptera) of Fennoscandia and Denmark. Fauna Entomol Scand. 1979;8:1–174.

[38] Seifert B (2007). Die Ameisen Mittel- und Nordeuropas.

[39] Bhatkar A, Whitcomb WH (1970). Artificial diet for rearing various species of ants. Fla Entomol.

[40] Folch J, Lees M, Stanley GHS (1957). A simple method for the isolation and purification of total lipides from animal tissues. J Biol Chem.

[41] Gómez-Brandón M, Lores M, Domínguez J (2010). A new combination of extraction and derivatization methods that reduces the complexity and preparation time in determining phospholipid fatty acids in solid environmental samples. Bioresour Technol.

[42] Frostegård Å, Tunlid A, Bååth E (1991). Microbial biomass measured as total lipid phosphate in soils of different organic content. J Microbiol Methods.

[43] Zelles L (1999). Fatty acid patterns of phospholipids and lipopolysaccharides in the characterisation of microbial communities in soil: a review. Biol Fertil Soils.

[44] Stein SE. Mass Spectra by NIST Mass Spec Data Center. In: Lindstrom PJ, Mallard WG, editors. NIST Chemistry WebBook. National Institute of Standards and Technology. 2015. http://webbook.nist.gov. Accessed 10 Oct 2016.

[45] Dunkelblum E, Tan SH, Silk PJ. Double-bond location in monounsaturated fatty acids by dimethyl disulfide derivatization and mass spectrometry: application to analysis of fatty acids in pheromone glands of four Lepidoptera. J Chem Ecol. 1985;11:265–77. 10.1007/BF01411414 24309959

[46] Pinheiro J, Bates D, DebRoy S, Sarkar D, R Core Team. nlme: Linear and nonlinear mixed effects models. 2016. http://CRAN.R-project.org/package=nlme. Accessed 15 Dec 2016.

[47] Hothorn T, Bretz F, Westfall P (2008). Simultaneous inference in general parametric models. Biom J.

[48] Anderson MJ (2001). A new method for non-parametric multivariate analysis of variance. Austral Ecol.

[49] Anderson MJ (2006). Distance-based tests for homogeneity of multivariate dispersions. Biometrics.

[50] Clarke KR, Gorley RN (2015). PRIMER v7: User Manual/Tutorial.

[51] Palarea-Albaladejo J, Martín-Fernández JA (2015). zCompositions — R package for multivariate imputation of left-censored data under a compositional approach. Chemom Intell Lab Syst.

[52] van den Boogaart KG, Tolosana R, Bren M. compositions: Compositional data analysis. 2014. https://CRAN.R-project.org/package=compositions. Accessed 15 Dec 2016.

[53] Oksanen F, Blanchet FG, Kindt R, Legendre P, Minchin PR, O’Hara RB, et al. vegan: Community ecology package. 2016. https://CRAN.R-project.org/package=vegan. Accessed 15 Dec 2016.

[54] Brückner A, Heethoff M (2017). A chemo-ecologists’ practical guide to compositional data analysis. Chemoecology.

[55] R Core Team. R: A language and environment for statistical computing. R Foundation for Statistical Computing. 2016. https://www.R-project.org/. Accessed 15 Dec 2016.

[56] Thompson SN (1973). A review and comparative characterization of the fatty acid compositions of seven insect orders. Comp Biochem Physiol Part B Comp Biochem.

[57] Hanson BJ, Cummins KW, Cargill AS, Lowry RR (1985). Lipid content, fatty acid composition, and the effect of diet on fats of aquatic insects. Comp. Biochem. Physiol. Part B Comp. Biochem.

[58] Stanley-Samuelson DW, Howard RW, Akre RD. Nutritional interactions revealed by tissue fatty acid profiles of an obligate myrmecophilous predator, *Microdon albicomatus*, and its prey, *Myrmica incompleta*, (Diptera: Syrphidae) (Hymenoptera: Formicidae). Ann Entomol Soc Am. 1990;83:1108–15.

[59] Barlow JS (1964). Fatty acids in some insect and spider fats. Can J Biochem.

[60] Brian MV, Blum MS (1969). The influence of *Myrmica* queen head extracts on larval growth. J Insect Physiol.

[61] Brian MV (1973). Caste control through worker attack in the ant *Myrmica*. Insect Soc.

[62] Bhulaidok S, Sihamala O, Shen L, Li D (2010). Nutritional and fatty acid profiles of sun-dried edible black ants (*Polyrhachis vicina* Roger). Maejo Int J Sci Technol.

[63] Blaul B, Steinbauer R, Merkl P, Merkl R, Tschochner H, Ruther J (2014). Oleic acid is a precursor of linoleic acid and the male sex pheromone in *Nasonia vitripennis*. Insect Biochem Mol Biol.

[64] Wheeler WM (1910). Ants - their structure, development and behavior.

[65] Rosell RC, Wheeler DE (1995). Storage function and ultrastructure of the adult fat body in workers of the ant *Camponotus festinatus* (Buckley) (Hymenoptera : Formicidae). Int J Insect Morphol Embryol.

[66] Wang YJ, Happ GM. Larval development during the nomadic phase of a nearctic army ant, *Neivamyrmex nigrescens* (Cresson) (Hymenoptera: Formicidae). Int J Insect Morphol Embryol. 1974;3:73–86.

[67] Sorensen AA, Busch TM, Vinson SB (1985). Control of food influx by temporal subcastes in the fire ant, *Solenopsis invicta*. Behav Ecol Sociobiol.

[68] Tschinkel WR (1998). Sociometry and sociogenesis of colonies of the harvester ant, *Pogonomyrmex badius*: worker characteristics in relation to colony size and season. Insect Soc.

[69] Silberman RE, Gordon D, Ingram KK (2016). Nutrient stores predict task behaviors in diverse ant species. Insect Soc.

[70] Brian MV (1974). Brood-rearing behaviour in small cultures of the ant *Myrmica rubra* L. Anim Behav.

[71] Bolton B. Bolton B. An online catalog of the ants of the world. 2016. http://antcat.org/. Accessed 27 Feb 2017.

[72] Kück P, Hita Garcia F, Misof B, Meusemann K. Improved phylogenetic analyses corroborate a plausible position of *Martialis heureka* in the ant tree of life. Gilbert MTP, editor. PLoS ONE. 2011;6:e21031. 10.1371/journal.pone.0021031 PMC3123331 21731644

[73] Hughes L, Westoby MT, Jurado E (1994). Convergence of elaiosomes and insect prey: evidence from ant foraging behaviour and fatty acid composition. Funct Ecol.

[74] Reifenrath K, Becker C, Poethke HJ (2012). Diaspore trait preferences of dispersing ants. J Chem Ecol.

