# Supplementary material for: Patterns and dynamics of neutral lipid fatty acids in ants – implications for ecological studies
Source: Front Zool. 2017 Jul 13;14:36. doi: 10.1186/s12983-017-0221-1 (PMC5508481; doi:10.1186/s12983-017-0221-1)
Supplement: Supplementary file 1 — Supplementary information. (PDF 511 kb) [file 12983_2017_221_MOESM1_ESM.pdf]

Patterns and dynamics of fatty acids as trophic markers in Formicidae – implications to study ant ecology

Supplementary information

S1 – Linear mixed-effect models with absolute amounts of NLFAs

S2 – Dry weight of ants during the experiment

S3 – Data transformations for linear mixed-effect models

S4 – Linear mixed model with *M. rubra* workers and larvae

S5 – Effect of larval growth in saturated NLFAs

S6 – Factor loadings for Principal Components Analyses

S1 – Linear mixed-effect models with absolute amounts of NLFAs

The absolute amounts of NLFAs increased with dry weight for all ants (Table 1). For workers, significant results and trend directions were the same for LMMs with absolute amounts and dry weight as a cofactor, and with relative amounts of NLFAs (main text). For larvae, again LMMs with time and dry weight yielded similar results, with the exception of absolute total NLFAs and C18:2n6, which did not changed with time, but increased with dry weight. As explained in the main text, this increase was small compared to growth of other body components, thus the relative total amount decreased. Since time and dry weight were correlated, but each factor influenced individual NLFAs in a different manner, some other relationships changed. The models with relative amounts in the main text are better to understand larvae dynamics overall, because they account for both factors in the same analysis (experimental time as a factor and variables standardized by dry weight).

Table 1 – Effects of dry weight, time and treatment on absolute total amount [µg] and individual amounts of fatty acids.

	**Total NLFAs**				**C16:0**				**C18:0**				**C18:1n9**				**C18:2n6**			
	df	F	trend	p	df	F	trend	p	df	F	trend	p	df	F	trend	p	df	F	trend	p
F. fusca (n=48)																				
Dry weight	1	7.91	↑	**0.007**	1	11.07	↑	**0.002**	1	13.64	↑	**< 0.001**	1	12.74	↑	**< 0.001**	1	3.27		0.077
Treatment	1	0.03		0.855	1	1.95		0.170	1	1.50		0.226	1	1.00		0.322	1	77.99		**< 0.001**
Time	1	5.71	↑	**0.021**	1	15.30	↑	**< 0.001**	1	16.33	↑	**< 0.001**	1	7.15	↑	**0.010**	1	12.66		**0.001**
Treatment x Time	1	0.52		0.472	1	0.20		0.650	1	0.61		0.438	1	0.01		0.929	1	15.81	$\uparrow_{high}$ $\downarrow_{low}$	**< 0.001**
Residuals	43				43				43				43				43			
M. rubra (n=48)																				
Dry weight	1	9.19	↑	**0.004**	1	5.46	↑	**0.024**	1	0.01		0.921	1	10.08	↑	**0.003**	1	0.08		0.768
Treatment	1	0.02		0.870	1	0.98		0.327	1	0.02		0.875	1	0.18		0.671	1	42.94	↑	**< 0.001**
Time	1	5.27	↓	**0.027**	1	5.26	↓	**0.027**	1	18.28	↓	**< 0.001**	1	5.38	↓	**0.020**	1	2.27		0.143
Treatment x Time	1	0.00		0.984	1	0.14		0.708	1	0.71		0.401	1	0.00		0.988	1	0.51		0.482
Residuals	43				43				43				43				43			
M. rubra larvae [A] (n=38)																				
Treatment	1	7.10	↑	**0.011**	1	0.33		0.567	1	3.83		0.059	1	4.37	↑	**0.044**	1	12.85	↑	**0.001**
Time	1	1.42		0.241	1	1.88		0.179	1	17.13	↓	**< 0.001**	1	12.50	↑	**0.001**	1	1.19		0.282
Treatment x Time	1	0.48		0.494	1	1.06		0.309	1	0.19		0.661	1	3.90		0.057	1	0.93		0.341
Residuals	33				33				33				33				33			
M. rubra larvae [B] (n=38)																				
Treatment	1	8.22	↑	**0.007**	1	0.31		0.577	1	2.91		0.098	1	5.27	↑	**0.028**	1	14.09	↑	**< 0.001**
Dry weight	1	6.48	↑	**0.016**	1	0.03		0.874	1	4.84	↓	**0.035**	1	23.72	↑	**< 0.001**	1	4.24	↑	**0.048**
Treatment x Dry weight	1	0.51		0.482	1	1.27		0.269	1	0.15		0.701	1	3.05		0.090	1	1.38		0.248
Residuals	33				33				33				33				33			

Results of linear mixed-effect models. Trend indicates the direction of significant effects ($p < 0.05$, in bold). For larvae, [A] = time as a factor, [B] = dry weight as a factor.

S2 – Dry weight of ants during the experiment

Overall, dry weight of ants did not differ between treatments (Table 2). It did not change also for workers, which do not grow in dimensions after emerging from pupae (although their total weight may change by accumulating or spending fat reserves). Larvae in general grew over time during the experiment (Fig. 1).

Table 2 – Effects of treatment and time on dry weight of ants during the experiment time

	dry weight [mg]			
	df	F	trend	p
F. fusca (n=48)				
Treatment	1	1.08		0.31
Time	1	0.26		0.61
Treatment x time	1	0.22		0.64
Residuals	44			
M. rubra (n=48)				
Treatment	1	0.01		0.97
Time	1	1.30		0.26
Treatment x time	1	3.90		0.055
Residuals	44			
M. rubra larvae (n=38)				
Treatment	1	1.77		0.25
Time	1	19.30	↑	**< 0.001**
Treatment x time	1	2.90		0.099
Residuals	33			

Results of linear mixed-effect models. Trends indicate the direction of significant effects ($p < 0.05$, in bold).

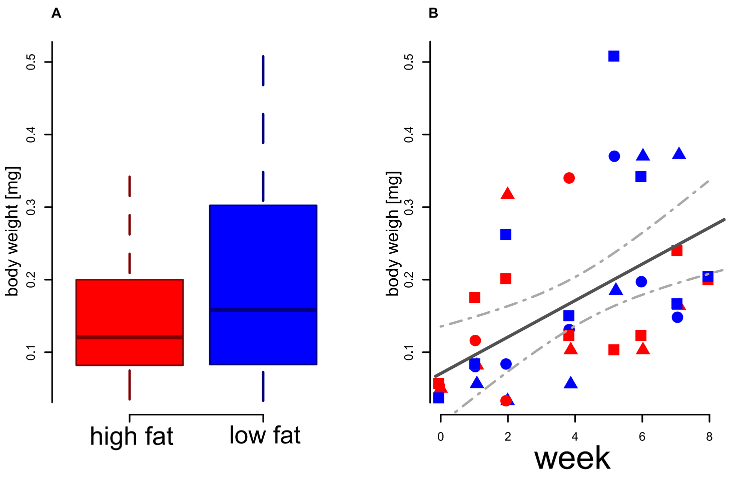

Fig. 1 – Increase in dry weight of *Myrmica rubra* larvae during the experiment.

S3 – Data transformations for linear mixed-effect models

Table 3 – Data transformations for linear mixed-effect models. For larvae, dry weight was also normalized by a square-root transformation (workers' weight had normal distributions and was not transformed).

	Total NLFAs	C16:0	C18:0	C18:1n9	C18:2n6
F. fusca	sqrt	log	-	log	log
M. rubra	sqrt	log	sqrt	sqrt	sqrt
M. rubra larvae	sqrt	sqrt	sqrt	log	log

sqrt = square-root transformation; log = logarithmic transformation; **-** = no transformation

S4 – Linear mixed model with *M. rubra* workers and larvae

	df	F	p
Treatment	1	2.30	0.148
Time	1	21.87	**< 0.001**
Life stage	1	22.14	**< 0.001**
Residuals	83		

S5 – Effect of larval growth in saturated NLFAs

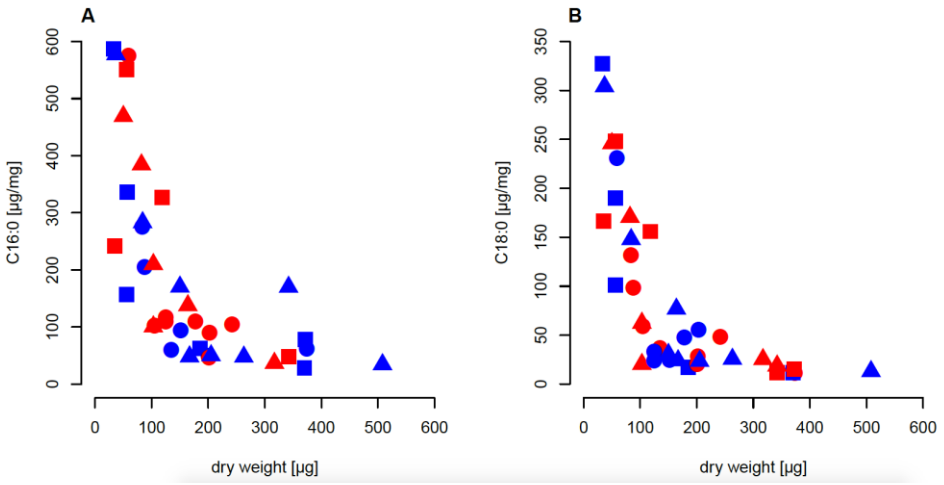

Fig. 2 – Decrease of relative amounts of saturated NLFAs with increasing body size in *M. rubra* larvae. Red = high-fat treatment. Blue = low-fat treatment.

S6 – Factor loadings for Principal Components Analyses

Formica fusca – main text, Fig. 5A

	PC1	PC2
C12:0	-1.1145	-0.2729
C14:0	-0.9414	-0.2072
C16:0	-0.1976	0.2512
C18:0	-0.2350	-0.1158
C20:0	-0.1617	-0.6004
C16:1n9	-0.7114	0.7445
C18:1n9	0.1906	0.7266
C18:2n6	0.5230	-0.7533
C18:3n3	1.4822	-0.1699
C18:3n6	1.1656	0.3972

Myrmica rubra – main text, Fig. 5B

	PC1	PC2
C12:0	-0.5150	-0.0592
C14:0	-0.6791	-0.0155
C16:0	-0.3305	-0.0970
C18:0	-0.5435	-0.3657
C20:0	-0.0761	-0.6645
C16:1n9	-0.8402	0.4124
C18:1n9	0.4310	1.0472
C18:2n6	0.4074	-0.0984
C18:3n3	1.6567	-0.2649
C18:3n6	0.4893	0.1057
